# Thalamo-cortical circuits during sensory attenuation in emerging psychosis: a combined magnetoencephalography and dynamic causal modelling study

**DOI:** 10.1038/s41537-023-00341-4

**Published:** 2023-04-28

**Authors:** Lingling Hua, Rick A. Adams, Tineke Grent-‘t-Jong, Ruchika Gajwani, Joachim Gross, Andrew I. Gumley, Rajeev Krishnadas, Stephen M. Lawrie, Frauke Schultze-Lutter, Matthias Schwannauer, Peter J. Uhlhaas

**Affiliations:** 1 Institute of Neuroscience and Psychology, University of Glasgow, Glasgow, UK; 2 Department of Psychiatry, The Affiliated Brain Hospital of Nanjing Medical University, 264 Guangzhou Road, Nanjing, 210029 China; 3 Centre for Medical Image Computing and AI, University College London, 90 High Holborn, London, WC1V 6LJ UK; 4 Max Planck-UCL Centre for Computational Psychiatry and Ageing Research, 10-12 Russell Square, London, WC1B 5EH UK; 5 Department of Child and Adolescent Psychiatry, Charité – Universitätsmedizin Berlin, corporate member of Freie Universität Berlin and Humboldt- Universität zu Berlin, Berlin, Germany; 6 Institute of Health and Wellbeing, University of Glasgow, Glasgow, UK; 7 Institute for Biomagnetism and Biosignalanalysis, University of Muenster, Muenster, Germany; 8 Department of Psychiatry, University of Edinburgh, Edinburgh, UK; 9 Department of Psychiatry and Psychotherapy, Medical Faculty, Heinrich Heine University, Düsseldorf, Germany; 10 Department of Psychology, Faculty of Psychology, Airlangga University, Surabaya, Indonesia; 11 University Hospital of Child and Adolescent Psychiatry and Psychotherapy, University of Bern, Bern, Switzerland; 12 Department of Clinical Psychology, University Edinburgh, Edinburgh, UK

**Keywords:** Biomarkers, Human behaviour

## Abstract

Evidence suggests that schizophrenia (ScZ) involves impairments in sensory attenuation. It is currently unclear, however, whether such deficits are present during early-stage psychosis as well as the underlying network and the potential as a biomarker. To address these questions, Magnetoencephalography (MEG) was used in combination with computational modeling to examine M100 responses that involved a “passive” condition during which tones were binaurally presented, while in an “active” condition participants were asked to generate a tone via a button press. MEG data were obtained from 109 clinical high-risk for psychosis (CHR-P) participants, 23 people with a first-episode psychosis (FEP), and 48 healthy controls (HC). M100 responses at sensor and source level in the left and right thalamus (THA), Heschl’s gyrus (HES), superior temporal gyrus (STG) and right inferior parietal cortex (IPL) were examined and dynamic causal modeling (DCM) was performed. Furthermore, the relationship between sensory attenuation and persistence of attenuated psychotic symptoms (APS) and transition to psychosis was investigated in CHR-P participants. Sensory attenuation was impaired in left HES, left STG and left THA in FEP patients, while in the CHR-P group deficits were observed only in right HES. DCM results revealed that CHR-P participants showed reduced top-down modulation from the right IPL to the right HES. Importantly, deficits in sensory attenuation did not predict clinical outcomes in the CHR-P group. Our results show that early-stage psychosis involves impaired sensory attenuation in auditory and thalamic regions but may not predict clinical outcomes in CHR-P participants.

## Introduction

Schizophrenia (ScZ) is typically a severe psychotic disorder that involves profound cognitive deficits and aberrant experiences of the self^1–3^. Previous theoretical formulations have proposed that self-disturbances constitute a fundamental aspect of ScZ that can be linked to several symptoms in which the sense of agency is disrupted, such as delusions of control as well as certain hallucinations^4,5^.

During normal brain functioning, the sense of agency is generated when predictions about the sensory feedback from a movement match with actual sensory feedback, leading to attenuation of the incoming stimulus (sensory attenuation)^6,7^. The greater the mismatch (i.e., prediction error), the more likely that sensations are inferred to be externally generated rather than self-generated. Studies with electro- and magnetoencephalography (EEG/MEG) have shown a reduction of the auditory N/M100 amplitude in self-generated tones vs external-generated tones^8,9^.

Cortical and subcortical regions contribute toward sensory attenuation and movement perception. Motor-related areas have been proposed to generate an efference copy to predict the sensory consequences of voluntary actions^10,11^, while the thalamus acts as a hub to propagate the prediction signal (efference copy) to higher-order cortical areas^12^. Furthermore, there is evidence that the parietal cortex may contribute towards sensory attenuation^13^ by integrating sensory and motor information^14^, while the cerebellum, in cooperation with the parietal cortex, plays a role in comparing predicted and forthcoming signals^13^. In addition, there is emerging data that bidirectional information flow between thalamus, IPL, and auditory cortex supports sensory attenuation during normal brain functioning^15^.

Several studies have provided evidence for the hypothesis that ScZ-patients are impaired in their ability to predict the sensory consequences of their actions^16^ which could underlie deficits in sensory attenuation^17–19^. A series of studies by Ford and colleagues have shown that patients with ScZ are characterized by impaired N100 sensory attenuation during self-generated speech^17,20^. Importantly, abnormalities in sensory attenuation have been observed across the psychosis spectrum^21–24^.

More recently, several studies have also examined the presence of sensory attenuation deficits in early-stage psychosis^25,26^. Patients with early-stage ScZ were characterized by deficient N100 suppression^25,26^. In contrast, EEG studies in participants meeting clinical high-risk for psychosis criteria (CHR-P)^27,28^ have shown intact sensory attenuation^27^, while there is also evidence for a deficit in CHR-Ps^25,26^.

In the current study, we aimed to extend the evidence on sensory attenuation dysfunctions in early-stage psychosis through a state-of-the-art MEG approach in combination with computational modeling to identify the brain regions and underlying networks involved in sensory attenuation. To this end, we recruited CHR-P participants, FEP patients, and a group of healthy controls (HC). In addition to the analysis of sensor-level data, we focussed on time-courses in auditory regions, thalamus, parietal and frontal areas based on our previous findings, followed by dynamic causal modeling (DCM) which allowed us to test different models of sensory attenuation deficits. In a second step, we investigated the possibility that impaired sensory attenuation would predict clinical outcomes in CHR-P participants, such as the persistence of attenuated psychotic symptoms (APS)^29^ and transition to psychosis^29,30^, as well as the relationship with cognitive deficits and functional impairments based on previous empirical and theoretical data^25,31–33^.

## Methods

### Participants

MEG data were collected as part of the baseline assessment in the Youth Mental Health Risk and Resilience Study (YouR-Study), funded by the Medical Research Council (MRC)^34^. A sample of 109 CHR-P, 48 HC, and 23 FEP participants were included in the analysis. CHR-P status was confirmed through the Comprehensive Assessment of At-Risk Mental States (CAARMS) interview^29^ and the Schizophrenia Proneness Instrument, Adult version (SPI-A)^35^. Participants were included in the CHR-P group when they met one or more of the following criteria: 1) Basic Symptom criteria according to the SPI-A instrument^36^ and/or 2) ultra-high-risk critera (UHR) according to CAARMS-interview^29^ which include APS, functional decline as well as a first-degree family history of a psychotic disorder and Brief Limited Intermittent Psychotic Symptoms (BLIPS).

Patients with FEP were included if (a) they reported the first treatment contact for a psychotic disorder and (b) met criteria for a psychotic disorder on the Structured Clinical Interview for DSM-V^36^. Current psychotic symptoms were assessed with the Positive and Negative Symptom Scale (PANSS)^37^. Neurocognition was assessed with the Brief Assessment of Cognition in Schizophrenia (BACS)^38^ in HCs and CHR-Ps only. The study was approved by the ethical committees of the University of Glasgow and the National Health Services Research Ethical Committee Glasgow and Greater Clyde. All participants provided written informed consent.

### Clinical follow-up

Participants meeting CHR-P criteria were reassessed at intervals of 3, 6, 9, 12, 18, 24, 30, and 36 months to examine persistence of CHR-P criteria and transition to psychosis. Persistence of CHR-P criteria was operationalized as the continued presence of APS up to 12 months.

### Experimental paradigm

**Fig. 1:**
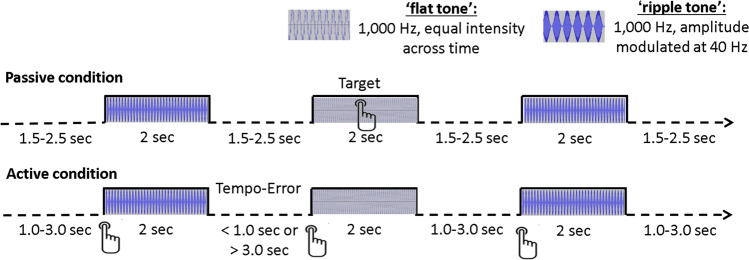
Experimental paradigm. In the passive condition, participants responded to 10 “flat” tones (1000 Hz, 2000 ms duration, 93 dB) and passively listened to 100 “ripple” tones (40 Hz amplitude-modulated 1000 Hz carrier tones, 2000 ms duration, 87 dB) with an average jittered stimulus-onset-asynchrony (SOA) of 4000 ms (3500–4500 ms). In the active condition, 100 ripple tones were elicited through a button press at ~ 4000 ms SOA (between 3000 and 5000 ms). A flat tone was presented in this condition when the response was outside the SOA.

All participants (*n* = 49 HC, *n* = 109 CHR-P, *n* = 23 FEP) underwent MEG recording during an auditory task (Fig. 1) that consisted of two blocks of trials, including two auditory stimuli, a 1000 Hz tone of constant intensity (‘flat’ tone, 2 sec duration, 93 dB), and a 40-Hz amplitude-modulated (AM) 1000 Hz tone (‘ripple’ tone, 2 sec duration, 87 dB). In the passive condition block (no agency), 100 ripple tones and 10 flat tones were binaurally delivered through plastic tubes with random inter-stimulus intervals between 1.5 and 2.5 sec. The stimulus-onset asynchrony (SOA) was between 3.5 sec and 5.5 sec.

Participants were instructed to press a button with their right index finger when a flat tone occurred and were asked to ignore the ripple tones. In the active (agency) condition block, participants were asked to press a button approximately every 4 sec, after which a ripple tone was presented. To ensure inter-trial intervals between the active and passive condition were similar, a flat tone was delivered if the participants pressed the button earlier than 3 sec or later than 5 sec. These trials were excluded from analysis. The task was terminated when 100 ripple tones were collected.

A motor-only condition was not included as source-level analyses were planned, which allowed adequate separation between contributions from motor regions and auditory regions to scalp-measured magnetic fields. The contrast of conditions (both including movements and tones) was designed to reveal the effects of agency on tone-perception areas.

### Neuroimaging data collection

All data were acquired on a 248-magnetometers whole-head MEG system (MAGNES 3,600 WH, 4-D Neuroimaging) with a sampling rate of 1017.25 Hz at the Center for Cognitive Neuroimaging, University of Glasgow. Prior to the MEG recording, the head-shape and five head position indicator coils (including nasion, bilateral pre-auricular points and two points on the left and right forehead) were digitized using a Polhemus Fastrack digitizer. Additionally, T1-weighted structural magnetic resonance images (MRIs) were obtained on a 3 Tesla scanner (Siemens, Tim Trio System) using a 3D Magnetization Prepared Rapid Gradient Echo sequence. The parameters were: 1 × 1 × 1 mm resolution, 192 volumes, TR = 2.250 ms, TE = 2.6 ms, FA = 9°.

### MEG data analysis

MEG data were analyzed with the open-source Fieldtrip Toolbox (Version 2018). Raw data were segmented from −1 to 3 sec, time-locked to the onset of ripple tone. Segments were subsequently filtered with a discrete Fourier transform filter at 50, 100, 150 Hz and then down-sampled to 300 Hz. Trials with SQUID jumps or large fluctuations artefacts were first removed by visual inspection. Faulty sensors with large signal variance or whose signals were flat were removed. Independent component analysis (ICA) and/or principal component analysis (PCA) were used to remove contributions from eye blinks and eye movements, as well as heartbeat and muscle activity. After cleaning, trials for the active condition were on average 94.1 ± 2.9 for HC group, 92.8 ± 5.6 for CHR-P group, and 92.5 ± 9.7 for the FEP group. For the passive condition, these numbers were 94.2 ± 3.0 for HC, 93.5 ± 2.5 for CHR-P, and 92.5 ± 3.3) for FEP group, respectively.

Prior to trial averaging, MEG data were band-pass filtered between 1 and 30 Hz with a two-pass Butterworth filter (filter order 6). Subsequently, all trials were baseline corrected, using a baseline window between −300 ms and −100 ms. Filtered sensor-level data were transformed from the axial-magnetometer to planar gradient signals^39^.

Individual T1-weighted MRIs were co-registered with MEG data using three anatomical landmarks (the nasion, right, and left pre-auricular points), followed by an automatic more fine-grained co-registration procedure with the ICP algorithm^40^. The co-registered MRI data were segmented into white matter, gray matter, and CSF before applying a single-shell volume conductor model to compute the individual head model. The lead fields were computed from the time-domain covariance matrix. A source model grid was based on a normalized individual MRI in a 6 mm template MRI (Montreal Neurological Institute, MNI).

A linearly constrained minimum variance (LCMV) beamformer^41^ was used to reconstruct virtual-channel source-space time-series data in 7 regions of interest (ROI) with a priori-defined central node MNI coordinates obtained from BrainNet Viewer software^42^. These ROIs were based on our previous paper^15^ that reported differences between active and passive conditions in the following regions in healthy controls: bilateral Heschl’ gyrus (LHES [−42, −19, 10], RHES [46, −17, 10]), bilateral superior temporal gyrus (LSTG [−53, −21, 7], RSTG [58, −22, 7]), bilateral THA (LTHA [−11, −18, 8], RTHA [13, −18, 8]), and right inferior parietal lobule (RIPL [46, −46, 50]).

Spatial filters were generated from covariance matrices, computed from unfiltered data across the entire epoch (−1 sec to 3 sec), and then applied to generate the time course of each trial in active and passive conditions separately. The covariance matrix was regularized by 5% of its eigenvalues to adjust the trade-off between spatial selectivity and sensitivity to uncorrelated sensor noise. Finally, the three orientation signals were combined using the first Singular Value Decomposition (SVD) component. The extracted time-series were subsequently filtered between 0.1 and 30 Hz, and baseline corrected between -300 ms and -100 ms, before averaging across trials.

### Statistics

For demographic, clinical, and neuropsychological variables, one-way Welch ANOVAs were used to test for group differences. BACS data were first Z-score standardized to HCs, while controlling for gender. Gender group differences were tested with Chi-Square tests. Alpha levels were 0.05 and two-sided. For post-hoc tests, Games-Howell corrected for multiple comparisons were employed.

At sensor-level, M100 amplitude differences between the active and passive condition (sensory attenuation effect) were tested between 90-140 ms based on visual inspection of the peak of the grand-average M100 amplitude across all participants. Group differences in sensory attenuation were examined with a cluster-based non-parametric Monte Carlo Permutation independent-sample *t* test^43^ (1000 permutations, alpha-level 0.05, two-tailed).

At source level, the analysis window for M100 sensory attenuation focussed on a timewindow between 90 and 150 ms, based on cluster-based non-parametric Monte Carlo Permutation^43^ dependent-sample t-tests (1000 permutations, alpha-level 0.05, two-tailed) estimated for M100 condition differences (Active vs Passive) across all 7 included ROIs and all participants (orthogonal contrast), using a search window between 50 ms and 200 ms post-stimulus-onset. Subsequently, data for each participant and each ROI was averaged between 90–150 ms and submitted to a mixed-design ANOVA, including the within-subject factor ROI (LHES, RHES, LSTG, RSTG, LTHA, RTHA, and RIPL) and the between-subject factor GROUP (HC, CHR-P, FEP). Post-hoc group effects were Games-Howell corrected for multiple comparisons. ROI effects were followed up by one-way Welch ANOVAs and further tested for group effects in significant ROIs, using bias-corrected and accelerated bootstrapping (*n* = 1000) and Games-Howell correction to control Type 1 errors.

A similar approach was used to test for M100 group differences in the active and passive conditions as well as for the relationship between sensory attenuation and clinical outcomes (persistence of APS, transition to psychosis). Finally, linear regression with backward selection were used to test associations between sensory attenuation in CHR-Ps in significant ROIs (RHES) and clinical features, including global assessment of functioning (GAF), total CAARMS severity and CAARMS subscale scores, total SPI-A severity, and BACS scores. CAARMS subscale scores included Unusual Thought Content (UTC), Non-Bizarre Ideas (NBI), Perceptual Abnormalities (PA), and Disorganized Speech (DS) scores multiplied by their frequency scores. Tolerance and the variance inflation factor (VIF) were estimated to identify multi-collinearity of independent variables.

### Dynamic causal modeling

DCM was used to examine network connectivity changes between thalamus, auditory regions, and parietal areas that could explain group differences in sensory attenuation effects. To simplify the DCM models, we omitted the bilateral STG and included only bilateral THA, bilateral HES, and RIPL.

Conceptually, ‘effective connectivity’ between neural nodes in DCM can be either structural or modulatory. Structural—forward, backward, and lateral connections—between nodes convey changes in brain activity elicited by a stimulus (i.e. a driving input). Modulatory connections parameterize the effects of (context-dependent) experimental factors on forward and backward (‘extrinsic’) connections, and can thus assess whether sensory attenuation is manifest in bottom-up or top-down message passing, or both. In addition, modulation of self-inhibitory connections (within each source) was permitted to test for ‘intrinsic’ changes in neural excitability^44^.

**Fig. 2:**
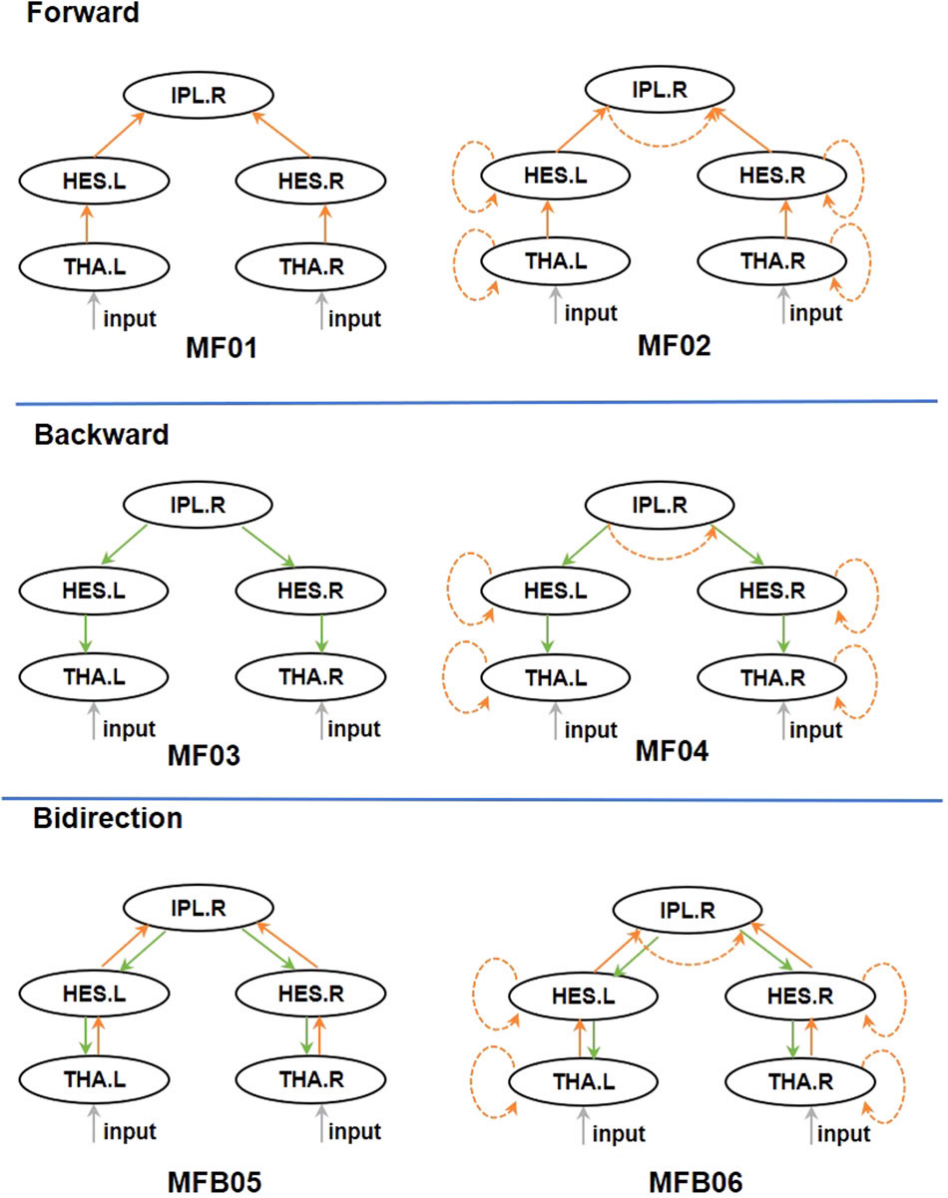
DCM model structure. Top panel: model structure for the Forward Family. Middle panel: model structure of the Backward Family. Bottom panel: model structure of the Bidirectional Family. The three families are furthermore subdivided by models with or without self-modulated connections (yellow dotted line). The gray line denotes the input via the thalamus. THA Thalamus, HES Heschl’s Gyrus, IPL Inferior Parietal Lobe, L left, R right.

DCM models were divided into three ‘families’ for all participants, in which either forward, backward, or bidirectional connections were used to model group differences. Each family contained one model with and one without modulatory intrinsic connections (Fig. 2). In addition, a null DCM model containing only intrinsic connections was used as comparison model. In total, seven models were constructed for all participants.

Auditory ERF responses between -100 ms and 200 ms were analyzed in the DCM pipeline using source-space virtual-channel data (using the ‘local field potential’ (LFP) option within DCM). Given that we were particularly interested in changes in connection strengths during sensory attenuation relative to a baseline condition (auditory input without sensory attenuation), the between-condition effects were set to 0 (baseline) and 1 (active). DCM was performed using Statistical Parametric Mapping 12 (SPM12, v7487) (https://www.fil.ion.ucl.ac.uk/spm/).

### DCM statistics: parametric empirical bayes (PEB)

PEB is a hierarchical Bayesian version of a general linear model (GLM) that can infer which connectivity parameters are similar or differ between groups^45^. The posterior parameter estimates (including posterior expectations and their covariances) estimated from the first level (Bayesian model inversion within subjects) were collated and modeled using this GLM^46^. The advantage of using PEB is that parameters estimated with the most precision (e.g., from the best-fitting models) contribute most to subsequent inferences about group differences.

A model space containing different combinations of forward, backward, and self-inhibitory connections was used to test for group differences in parameters.

## Results

### Demographic and neuropsychological data

The groups differed in sex composition (Table 1; *p* = 0.004), with the FEP group being characterized by significantly more males than the HC (*p* = 0.017) and CHR-P groups (*p* < 0.001). CHR-Ps had significantly fewer years of education than HCs (*p* = 0.021). GAF scores were significantly different across groups (*p* < 0.001), with the FEP group having the lowest GAF scores. Finally, CHR-P participants were significantly impaired relative to controls in BACS token motor task (*p* < 0.001), symbol coding task (*p* = 0.004), and composite scores (*p* = 0.002).

**Table 1:** Demographics, general cognition, and clinical data.

	HC	CHR-P	FEP	Group effect^a^	Post-hoc comparisons
Number of participants	48	109	23		
Age: years (SD)	22.8 (3.6)	22.0 (4.5)	23.2 (3.5)	F(2,62.6) = 1.2 *p* = 0.31	
Gender: male/female (% male)	15/33 (31.2)	28/81 (25.7)	14/9 (60.9)	χ(2) = 10.9, *p* = 0.004	FEP > HC: *p* = 0.017 FEP > CHR-P: *p* < 0.001
Education: years (SD)	16.7 (3.0)	15.3 (3.1)	15.4 (3.1)	F(2,56.4) = 3.8, *p* = 0.029	CHR-P < HC: *p* = 0.021
BACS^b^: mean (SD)					
Verbal memory	52 (8.3)	-0.33 (1.3)	NA	Not significant	
Digit sequencing	21 (2.7)	-0.08 (1.4)	NA	Not significant	
Token motor	81 (11.4)	-1.03 (1.3)	NA	F(1,117.3) = 31.3, *p* < 0.001	CHR-P < HC
Verbal fluency	58 (13.2)	0.01 (1.3)	NA	Not significant	
Symbol coding	74 (11.9)	-0.54 (1.1)	NA	F(1,101.7) = 8.7, *p* = 0.004	CHR-P < HC
Tower of London	19 (1.8)	-0.16 (1.4)	NA	Not significant	
Composite score	305 (24.4)	-0.57 (1.4)	NA	F(1,121.9) = 9.6, *p* = 0.002	CHR-P < HC
CAARMS scores^c^: mean (SD)					
Unusual Thought Content	0.0 (0.1)	5.6 (7.5)	NA		
Non-bizarre Ideas	0.3 (1.7)	10.1 (8.5)	NA		
Perceptual Abnormalities	0.3 (1.1)	8.7 (6.4)	NA		
Disorganized Speech	0.0 (0.3)	4.6 (5.4)	NA		
Total CAARMS severity	0.7 (2.4)	29.0 (18.3)	NA		
SPI-A severity^d^: mean (SD)	0.0	11.2 (11.9)	NA		
GAF: mean (SD)	87.8 (6.4)	57.7 (13.7)	39.5 (14.6)	F(2,56.0) = 246.3, *p* < 0.001	FEP < HC: *p* < 0.001 CHR-P < HC: *p* < 0.001 FEP < CHR-P: *p* < 0.001
GF-role: mean (SD)	8.6 (0.8)	7.4 (1.2)	NA		
GF-social: mean (SD)	8.8 (0.4)	7.5 (1.2)	NA		
PANSS: mean (SD)					
Positive	NA	NA	17.9 (7.0)		
Negative	NA	NA	14.7 (8.6)		
Cognitive	NA	NA	19.8 (9.2)		
Excitement	NA	NA	8.6 (4.6)		
Depression	NA	NA	11.4 (5.8)		
Total score	NA	NA	72.4 (27.8)		
DSM-IV/SCID-IP					
Diagnostic categories of FEP group participants	Schizophrenia (*n* = 4), Schizophreniform disorder (*n* = 3), Psychotic Disorder NOS (*n* = 7), Mood Disorders with Psychotic Symptoms (*n* = 7), Schizoaffective disorder (*n* = 1), Delusional Disorder (*n* = 1)				
Medication^e^: *n* (%)					
None	48 (100)	57 (52)	4 (17)		
Anti-depressants	0	42 (39)	12 (52)		
Mood stabilizers	0	5 (5)	0		
Anti-psychotics	0	2 (2)	12 (52)		
Other	1 (2)	18 (17)	6 (26)		

*BACS* Brief Assessment of Cognition in Schizophrenia, *CAARMS* Comprehensive Assessment of At-Risk Mental States, *HC* healthy controls, *CHR-P* clinical high-risk for psychosis, *FEP* first-episode psychosis, *GAF* global assessment of functioning, *GF* global functioning, *PANSS* Positive and Negative Symptom Scale, *SPI-A* Schizophrenia Proneness Instrument, Adult version, *SD* standard deviation of the mean *NA* not assessed.

^a^Except for ‘gender’ statistical testing, which is based on Chi-Square tests, all other tests are based on one-way Welch ANOVAs (alpha = 0.05, two-sided, post-hoc Games-Howell-corrected for multiple comparisons).

^b^BACS scores for CHR-P group were standardized to control group data, controlled for gender. HC group data represents raw scores.

^c^CAARMS scores: entries represent global scores (max 6) multiplied by frequency scores (max 6).

^d^SPI-A severity scores: entries represent sum score of all items.

^e^Multiple ratings possible.

### Follow-up outcomes

Follow-up CAARMS data were available for 103 of the 109 CHR-P participants of which *n* = 70 met APS criteria at baseline. After 12 months follow-up, *n* = 34 CHR-P participants continued to meet APS criteria (persistent APS-P group), whereas *n* = 36 were remitted (non-persistent APS-NP group). Compared to the APS-NP group, the APS-P group scored significantly higher on CAARMs total symptoms (Table 2) as well as higher scores on the UTC and NBI-subscales. 10 CHR-P participants converted to a FEP (CHR-P-T) (Table 2). Average time of transitioning to psychosis was 17.6 months in CHR-P group.

**Table 2:** Demographic and clinical characteristics: follow-up CHR-P participants.

	APS non-persistent	APS persistent	Converters	Pairwise comparison^a^
Number of participants	36	34	10	
Age, years (SD)	21.8 (4.3)	23.0 (5.2)	20.7 (5.1)	Not significant
Sex, male/female (%male)	7/29 (19.4)	11/23 (32.3)	1/9 (10.0)	Not significant
Education, years (SD)	15.5 (3.0)	15.5 (3.6)	14.3 (2.2)	Not significant
BACS^b^, mean (SD)				
Verbal memory	−0.25 (1.2)	−0.47 (1.2)	−0.50 (1.0)	Not significant
Digit sequencing	−0.05 (1.5)	−0.42 (1.6)	−0.36 (0.9)	Not significant
Token motor	−0.94 (1.2)	−1.31 (1.3)	−0.88 (1.3)	Not significant
Verbal fluency	0.06 (1.4)	0.05 (1.5)	−0.15 (0.7)	Not significant
Symbol coding	−0.49 (1.2)	−0.64 (0.9)	−0.84 (0.7)	Not significant
Tower of London	−0.35 (1.5)	−0.10 (1.1)	0.41 (1.2)	Not significant
Composite score	−0.56 (1.6)	−0.77 (1.4)	−0.64 (0.7)	Not significant
CAARMS, mean (SD)				
Unusual thought content	4.3 (6.1)	8.7 (8.8)	10.5 (9.8)	APS-P > APS-NP F(1,58.0) = 5.9, *p* = 0.018
Non-bizarre ideas	10.3 (8.4)	15.1 (7.7)	11.0 (7.2)	APS-P > APS-NP F(1,67.9) = 6.2, *p* = 0.015
Perceptual abnormalities	9.8 (5.8)	11.2 (7.0)	8.7 (6.5)	Not significant
Disorganized speech	4.4 (5.9)	6.1 (6.0)	5.4 (5.5)	Not significant
Total severity score	28.8 (14.5)	41.1 (16.1)	35.6 (19.0)	APS-P > APS-NP F(1,66.3) = 11.3, *p* = 0.001
SPI-A severity, mean (SD)	8.9 (11.1)	13.3 (16.1)	13.6 (11.4)	Not significant
GAF, mean (SD)	57.0 (14.2)	53.5 (13.6)	51.5 (6.9)	Not significant
GF-role, mean (SD)	7.5 (1.0)	7.1 (1.1)	7.2 (1.2)	Not significant
GF-social, mean (SD)	7.4 (1.1)	7.4 (1.4)	7.0 (0.9)	Not significant
Medication^c^				
None	18	19	4	
Anti-depressants	13	14	4	
Mood stabilizers	3	2	0	
Anti-psychotics	1	1	0	
Other (Unknown)	6	4	2	
Converters	Includes 1 converter who transitioned after 12 months	Includes 3 converters who transitioned after 12 months	Duration of transitioning after baseline recordings: 17.6 months (SD = 9.6)	Schizoaffective disorder with psychotic features (*n* = 3), psychotic disorder NOS (*n* = 3), mood disorder with psychotic features (*n* = 2), delusional disorder (*n* = 2)

*APS* attenuated psychotic symptoms, Persistent (APS-P) vs Non-persistent (APS-NP) CHR-Ps; *BACS* Brief Assessment of Cognition in Schizophrenia, *CAARMS* comprehensive assessment of at-risk mental states, *GAF* global assessment of functioning, *GF* global functioning, *SPI-A* schizophrenia proneness instrument, adult version, *SD* standard deviation of the mean.

^a^All *F* tests are Welch based; alpha = 0.05, two-sided, and do not include the converters, only the APS-P and APS-NP groups.

^b^BACS scores for clinical groups were standardized to control group data, controlled for gender category.

^c^If multiple medications were reported, they were scored separately in the different categories listed.

### Sensory attenuation effect at sensor level

**Fig. 3:**
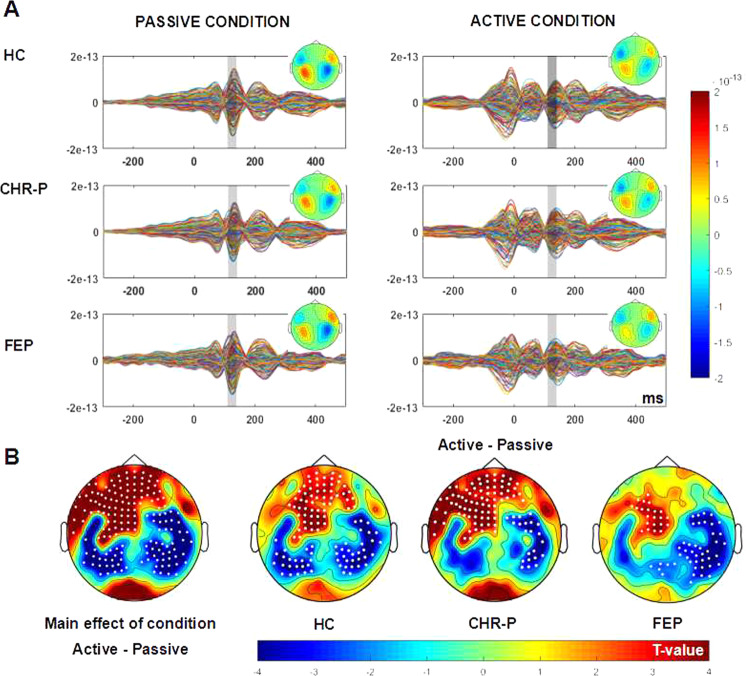
Sensor level sensory attenuation effects. **A** Grand-average butterfly, baseline corrected (−300 and −100 ms), ERF-time series were computed for all 248 sensors in 49 HCs, 109 CHR, and 23 FEP. Overlayed on the ERFs traces are topographical distribution plots of the M100 component in axial-magnetometer representation, plotted for data averaged over the 110–140 ms latency window. The area highlighted in gray indicates the sensory attenuation effect. **B** Topographical distributions in planar-magnetometer representation *t* values for the SAP effect across and within groups. Significant clusters of sensors (*p* < 0.05, two-tailed) are highlighted with white dots. HC healthy controls, CHR-P clinical high-risk psychosis, FEP first-episode psychosis.

The available average number of trials did not differ between groups (*p* > 0.05). There was a significant M100 amplitude difference across participants between active and passive conditions over frontal sensors (cluster-t(181) = 407.4, *p* = 0.002, 95% CI = [-0.0008,0.0048]) and temporal-parietal sensors (cluster1-t(181) = −191.4, *p* = 0.0020, CI = [−0.0008,0.0048]); cluster2-t(181) = −94-6, *p* = 0.018, CI = [0.0098 0.0262]) (Fig. 3). HCs had a significant sensory attenuation effect over frontal sensors (one cluster: t(47)=188.5, *p* = 0.002) and bilateral temporal-parietal sensors (cluster1: t(47) = −100.3, *p* = 0.012); cluster2: t(47) = −67.6, *p* = 0.028). In the CHR-P group, condition differences were also localized over frontal sensors (one cluster: t(109) = 295.7, *p* = 0.002), but across temporal-parietal sensors only the right hemisphere was characterized by a significant sensory attenuation effect (one cluster: t(109) = −70.6, *p* = 0.036). In the FEP group, significant differences between conditions were found over a smaller cluster of frontal sensors (one cluster: t(23) = 69.8, *p* = 0.034) and right temporal-parietal sensors (one cluster: t(23) = −140.4, *p* = 0.006). However, there were no statistically significant group difference in sensory attenuation as well as no significant group differences in M100 amplitude in either active or passive conditions (*p* > 0.05).

### Sensory attenuation effect at source level

**Fig. 4:**
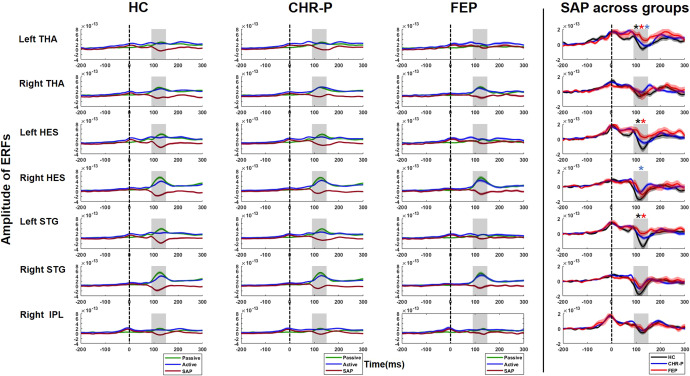
Source-level sensory attenuation effects. Source-reconstructed grand-average virtual-channel ERF traces for the 7 ROIs (left and right THA, HES, STG, and right IPL) for each group (HC, CHR-P, FEP). Right column: sensory attenuation effects per group. Black asterisk indicates a significant sensory attenuation effect in HC (*n* = 49), red in FEPs (*n* = 23) and blue in CHR-Ps (*n* = 109). The statistically significant *p* value was 0.05. THA Thalamus, HES Heschl’s Gyrus, STG superior temporal gyrus, HC healthy controls, CHR-P clinical high-risk psychosis, FEP, first-episode psychosis, SAP sensory attenuation.

Mixed-design ANOVAs (7 ROIs by 3 GROUPs) revealed a main effect of ROI (F(1,177) = 7.2, *p* = 0.008, partial *ɳ*^2^ = 0.039) and a main effect of GROUP (F(2,177) = 4.8, *p* = 0.009, partial *ɳ*^2^ = 0.052), with a trend toward an interaction effect between ROI and GROUP (F(2,177) = 2.4, *p* = 0.097, partial *ɳ*^2^ = 0.052). Post-hoc testing of the GROUP effect revealed significant differences across ROIs between HC and CHR-P (*p* = 0.019), HC and FEP (*p* = 0.015), and between CHR-P and FEP groups (*p* = 0.019). Furthermore, one-way Welch ANOVAs of the ROI effect revealed significant GROUP differences in LHES (F(2,64.7) = 4.4, *p* = 0.016), LSTG (F(2,64.0) = 4.5, *p* = 0.015), and LTHA (F(2,61.9) = 4.6, *p* = 0.013) and a trend in RHES (F(2,57.7) = 3.0, *p* = 0.059)(SI Table 1). Post-hoc pairwise comparisons revealed that this resulted from significantly lower sensory attenuation effects in FEP patients in LHES (*p* = 0.013, Hedges’ *g* = 0.67), LSTG (*p* = 0.01, *g* = 0.65), and LTHA (*p* = 0.012, *g* = 0.79), and significantly impaired sensory attenuation in CHR-Ps in RHES (*p* = 0.044, *g* = 0.42), compared to HCs (Fig. 4). Finally, there were no effects of gender on sensory attenuation effects nor were there differences between FEP patients who were treated with antipsychotic medication (APMs) versus those who were APM-naive.

### M100 sensory attenuation and clinical outcomes in CHR-P

A significant main effect of ROI (F(1,115) = 9.7, *p* = 0.002, partial *ɳ*^2^ = 0.078) and main effect of GROUP F(2,115) = 4.3, *p* = 0.016, partial *ɳ*^2^ = 0.069) was found, with post-hoc GROUP effects for HCs vs APS-NP (*n* = 36) (*p* = 0.028) and HCs vs APS-P (*n* = 34) (*p* = 0.058) across ROIs, but no differences between APS-NP and APS-P groups (SI: Figure 1).

For analyses comparing CHR-P-T (*n* = 10) vs. non-transitioned CHR-Ps (CHR-P-NT) (*n* = 93), a significant main effect of ROI (F(1,148) = 9.7, *p* = 0.001, partial *ɳ*^2^ = 0.070) and main effect of GROUP F(2,148) = 4.3, *p* = 0.03, partial *ɳ*^2^ = 0.046) was found, with post-hoc GROUP effects for HC vs CHR-P NT (*p* = 0.018) and, but no differences between CHR-P T vs. CHR-P NT groups (*p* = 0.97) (SI: Figure 2).

### M100 amplitude in the passive and active conditions

While there were no GROUP effects in the active condition, there was an effect in the passive condition. Specifically, a main effect of ROI (F(1,177) = 76.5, *p* < 0.001, partial *ɳ*^2^ = 0.302), a main effect of GROUP (F(2,177) = 3.4, *p* = 0.035, partial *ɳ*^2^ = 0.037), and a ROI * GROUP interaction effect (F(2,177) = 9.4, *p* = 0.003, partial *ɳ*^2^ = 0.064) was found. Post-hoc pairwise comparisons indicated that this resulted from a main effect across ROIs between HCs and FEP patients (*p* = 0.027). Within ROIs, GROUP effects were found for the LHES (F(2,77.7) = 18.4, *p* < 0.001), the LSTG (F(2,78.4) = 21.2, *p* < 0.001), and the LTHA ROI (F(2,72.7) = 12.1, *p* < 0.001). Post-hoc pairwise analyses showed that FEP patients had significantly lower M100 amplitudes in the passive condition compared to HCs and CHR-Ps in LHES (HC-vs-FEP: *p* = 0.001, *g* = 0.91; CHR-P-vs-FEP: *p* = 0.001, *g* = 0.79), LSTG (HC-vs-FEP: *p* < 0.001, *g* = 0.96; CHR-P-vs-FEP: *p* < 0.001, *g* = 0.83), and LTHA (HC-vs-FEP: *p* < 0.001, *g* = 0.92; CHR-P-vs-FEP: *p* = 0.001, *g* = 0.64) (SI Table 2).

### Correlations between sensory attenuation, psychopathology, and cognition

Linear regression analysis with backward selection of predictors (GAF, CAARMS severity, SPI-A severity, CAARMS subscales, and BACS scores), on sensory attenuation effects in the CHR-Ps in RHES revealed a significant negative correlation between sensory attenuation in RHES and BACS digit sequencing task (model F(1,107) = 15.7, *p* < 0.001, standardized beta = −0.358, *t* = −4.0, *p* < 0.001).

### DCM results

#### Connection parameter comparison between groups with PEB

**Fig. 5:**
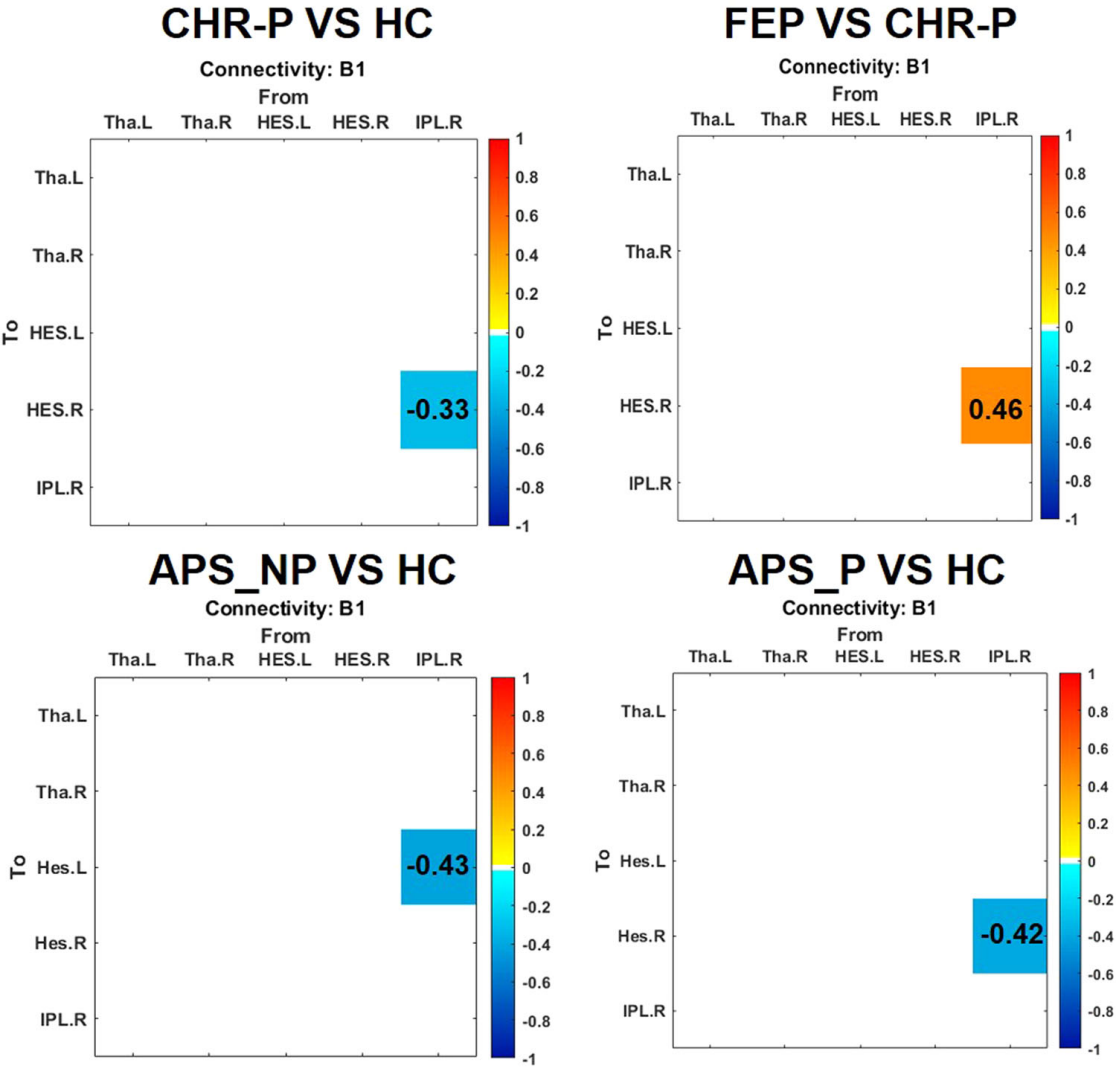
Group-level GLM parameters and BMA of parameters. Left panel: parameters of group-level Bayesian GLM between groups. The modulatory connection strength (Expected value) with more than 95% posterior probability is highlighted. Decreased connection strength was plotted in blue and increased connection strength in orange. PEB parametric empirical Bayesian, HC healthy controls, CHR-P clinical high-risk psychosis, FEP first-episode psychosis.

The PEB analysis (Fig. 5) revealed that CHR-P participants displayed reduced connection strength from the right IPL to the right HES with more than 95% posterior probability in comparison to HC and FEP participants. In contrast, no significant differences were observed between the FEP group and HC. In addition, the APS-NP group showed reduced connection strength from the right IPL to the left HES and the right HES when compared to HC group. There were no differences of >95% probability between APS-P and HCs, however. The CHR-P-NC group also showed reduced backward connectivity from right IPL to bilateral HES, and also from left HES to left THA.

## Discussion

This study examined deficits in auditory sensory attenuation in early-stage psychosis, the underlying network involved, and the relationship to clinical outcomes in CHR-P participants. We addressed these questions through a novel approach combining source-reconstructed MEG data with computational modeling. The results indicate that sensory attenuation was reduced in CHR-P participants and in FEP patients in an extended network involving left HES, left STG, and left THA. In addition, the sensory attenuation network in CHR-Ps was characterized by impaired top-down modulation of the auditory cortex.

Previous studies using EEG had provided conflicting evidence on whether sensory attenuation is reduced in CHR-P participants^25–28^ while there is consistent data for impaired sensory attenuation in ScZ^24,47^. In the current study, sensor-level responses in both FEP and CHR-P groups were intact compared to controls while virtual-channel data revealed decreased sensory attenuation that differed across illness stages. Specifically, CHR-P participants displayed reduced sensory attenuation in right HES while in the FEP group reductions were found in the left hemisphere comprising HES, STG, and THA. Accordingly, these data highlight the potential of source-reconstruction methods to identify and differentiate the contribution of brain regions towards impaired sensory attenuation in early-stage psychosis.

In contrast to a recent report^25^, we observed that the degree of sensory attenuation deficits was significantly related to illness stages. While CHR-P participants had a circumscribed impairment in right HES, the FEP group was characterized by more widespread impairments that preferentially targeted the left hemisphere and overall sensory attenuation deficits were more pronounced. Specifically, sensory attenuation deficits involved significant reductions in left HES, STG, and THA. Accordingly, these data suggest the possibility that sensory attenuation networks undergo progressive modifications during early-stage psychosis.

In particular, the involvement of the left hemisphere could indicate that sensory attenuation impairments in the FEP group could underlie the emergence of auditory hallucinations, a prominent feature in FEP patients^48^. However, the interpretation of the reduction in sensory attenuation in the FEP group is complicated by the fact that the M100 amplitude in the passive was significantly decreased as well, suggesting a deficit in the processing of bottom-up inputs in auditory areas that could contribute towards sensory attenuation impairments in FEP patients.

Computational modeling using a DCM approach revealed insights into effective connectivity underlying sensory attenuation dysfunction in early-stage psychosis. Reduced sensory attenuation in the right HES in CHR-P participants was explained by decreased backward connectivity from the right IPL to the right HES. Evidence suggests that the IPL plays an important role through interactions with the cerebellum^14^ in the prediction of motor outcomes^13^ and the backward connection from right IPL to HES was previously shown to be strengthened in controls during sensory attenuation in the same paradigm^15^. In the FEP group, however, the loss of sensory attenuation in the left hemisphere was not captured by the model. Instead, increased backward connectivity from right IPL to right HES relative to CHR-Ps was observed which could suggest a compensatory process for a left hemisphere deficit.

We also investigated the relationship between sensory attenuation and clinical outcomes in the CHR-P group. The identification of prognostic biomarkers for predicting clinical trajectories is of particular importance given that CHR-P participants constitute a highly heterogeneous population in terms of clinical trajectories^49^, with only a minority developing a persistent psychotic disorder^50^. In the current study, sensory attenuation deficits did not predict the transition to psychosis nor the persistence of APS in CHR-P participants.

These data are consistent with a previous EEG-study^25^ that also failed to observe a relationship between transition to psychosis and impairments in sensory attenuation in CHR-Ps. Accordingly, these data suggest that other electrophysiological biomarkers, such as gamma-band oscillations^51^ as well as certain ERPs/ERFs (MMN, P300)^52,53^ may have greater potential for dissecting the clinical heterogeneity associated with CHR-P criteria while sensory attenuation could potentially allow important insights into the brain mechanisms underlying self-disturbances during early-stage psychosis.

There are several limitations of our study. Firstly, the detection of thalamic activity with MEG remains challenging. However, emerging evidence supports the ability of MEG to detect activity in deeper brain areas, such as the thalamus^54,55^ and hippocampus^56^. In addition, we did not include a motor-only condition as a baseline for the sensorimotor system. This is because previous studies showed that sensory attenuation remains present after ruling out the motor contamination by subtracting motor activity from motor-auditory activity^57,58^.

In addition, the DCM analysis only compromised a subsection of brain regions that showed sensory attenuation effects. We intentionally selected only the HES, IPL, and thalamus since a larger number of sources would have increased the complexity of the DCM model significantly. Moreover, we did not include motor regions as indicated above as the driving input for both experimental conditions needs to be similar in DCM. Finally, the sample size in each group was significantly different, especially between the FEP and CHR-P groups, which could have impacted the results.

In summary, the current findings highlight that sensory attenuation is impaired in both CHR-P and FEPs. However, the networks and brain regions underlying sensory attenuation deficits in both groups were distinct, suggesting the involvement of right primary auditory cortex in the CHR-P group that correlated with cognitive deficits while in FEP patients, an extended network comprising left thalamus and auditory areas contributed towards sensory attenuation. Computational modelling indicated that sensory attenuation impairments in the CHR-P group involved reduced backward connectivity from right parietal to auditory cortex related to loss of backward connectivity from right parietal to auditory cortex. Importantly, we could not detect a relationship between clinical outcomes in CHR-P participants and the extent of sensory attenuation deficits. Together these data suggest that sensory attenuation may advance the understanding of core research into cognitive and physiological processes involved in early-stage psychosis.

## Supplementary information

SI Information

## Data Availability

MEG and clinical data as well as all relevant analysis scripts are available from the corresponding author upon request.
