# Supplementary material for: Thalamo-cortical circuits during sensory attenuation in emerging psychosis: a combined magnetoencephalography and dynamic causal modelling study
Source: Schizophrenia (Heidelb). 2023 Apr 28;9(1):25. doi: 10.1038/s41537-023-00341-4 (PMC10147678; doi:10.1038/s41537-023-00341-4)
Supplement: Supplementary file 1 — SI Information [file 41537_2023_341_MOESM1_ESM.docx]

Supplementary Figure 1. Differences between Active and Passive conditions in APS-P and APS-NP groups at 12 months


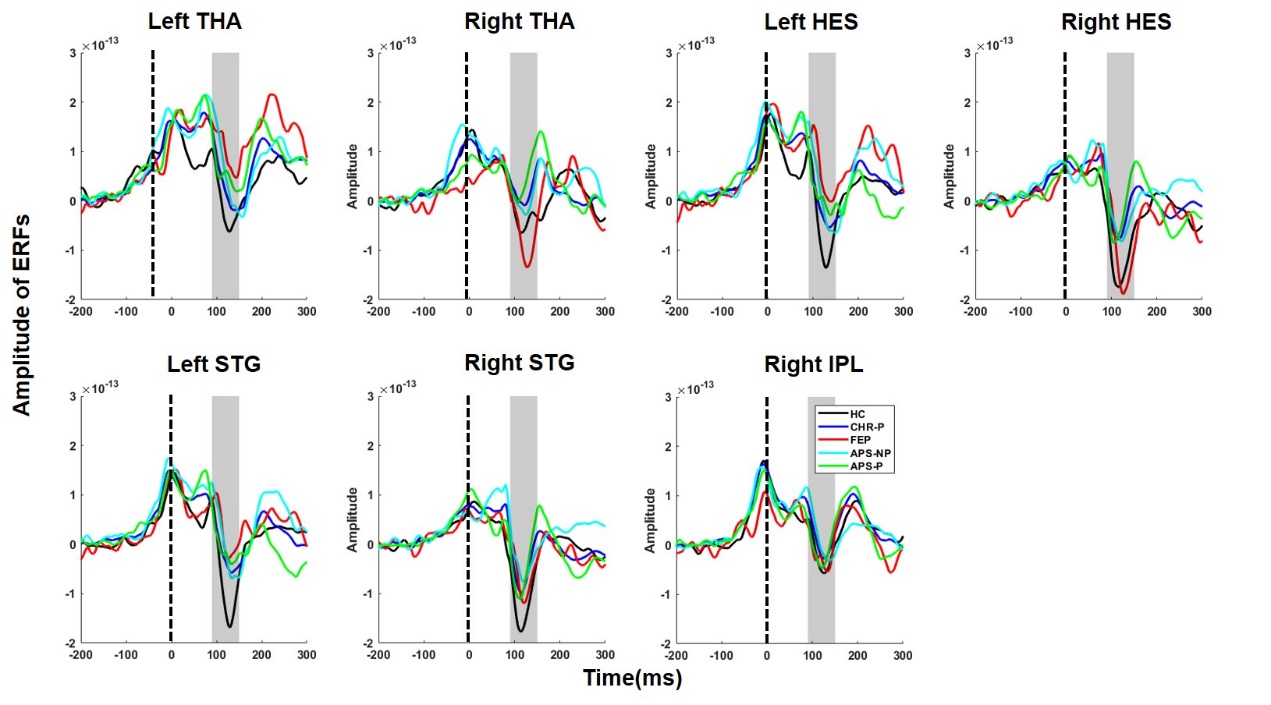


ERF traces in the active and passive conditions as well as the difference between two conditions in 7 ROIs per group (HC(n=49): black, CHR(n=109): blue, FEP(n=23): red; APS-NP(n=36): green; APS-P(n=34): magenta). The time window of interest (90-150 ms) is highlighted. Abbreviations: HES: Heschl’s Gyrus; STG: Superior Temporal Gyrus; THA: Thalamus; IPL: Inferior Parietal Cortex; HC: Healthy Controls; CHR-P: Clinical High-risk Psychosis; FEP: First-episode Psychosis.

Supplementary Figure 2. Differences between the Active and Passive Conditions in CHR-P-C and CHR-P-NC groups


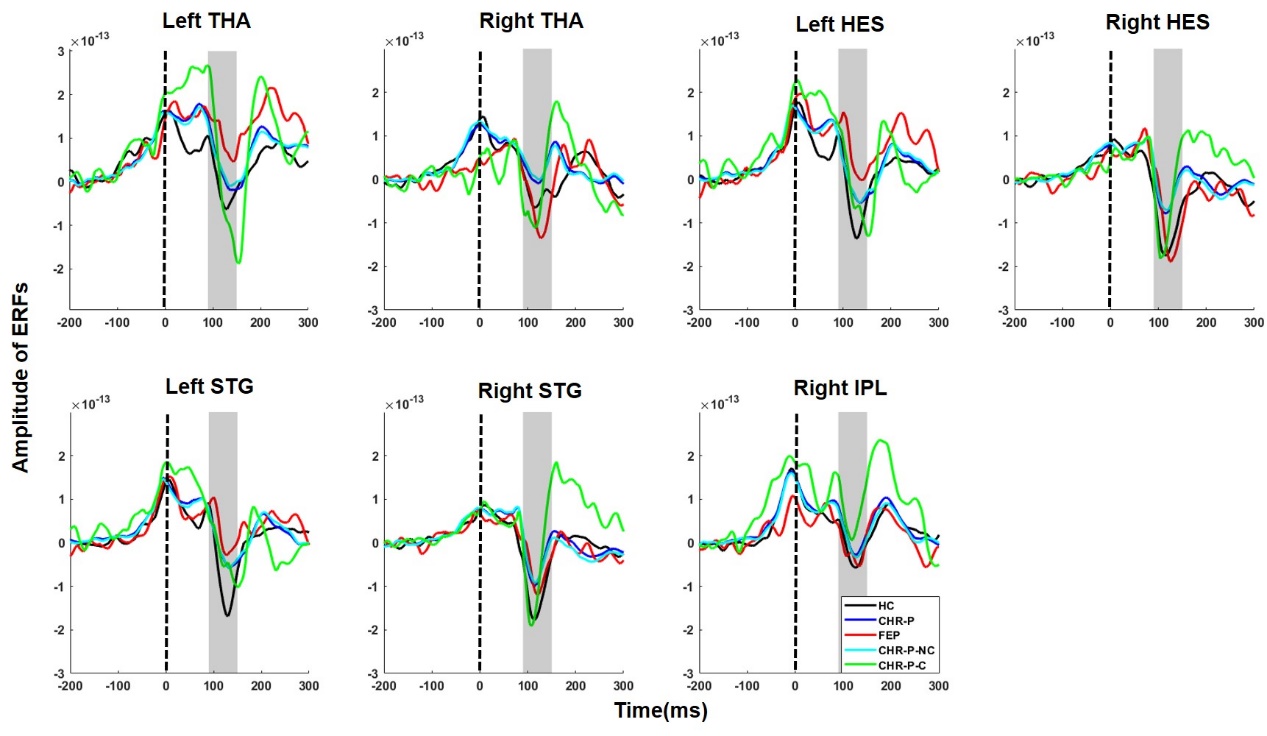


ERF traces in the active and passive conditions as well as the difference between two conditions in 7 ROIs per group (HC(n=49): black, CHR(n=109): blue, FEP(n=23): red; CHR-P-NC(n=99): green; CHR-P-C(n=10): magenta). The time window of interest (90-150 ms) is highlighted. The red asterisks indicated the group difference between HC and CHR-P-NC. Abbreviations: HES: Heschl’s Gyrus; STG: Superior Temporal Gyrus; THA: Thalamus; IPL: Inferior Parietal Cortex; HC: Healthy Controls; CHR-P: Clinical High-risk Psychosis; FEP: First-episode Psychosis.

Supplementary Table 1. Sensory attenuation effect at the source level.


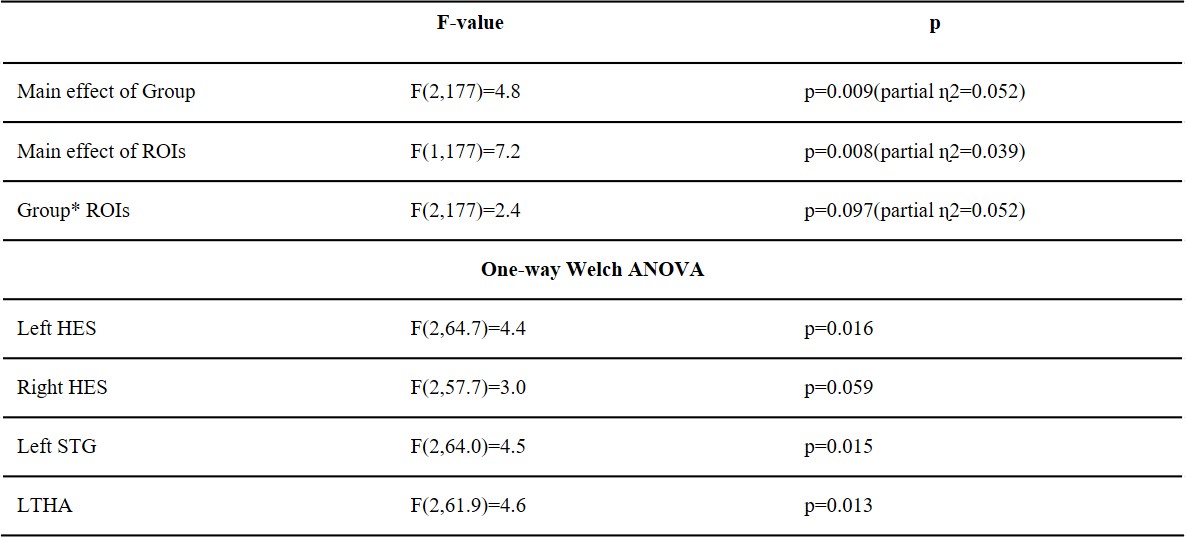


Abbreviations: HES: Heschl’s Gyrus; STG: Superior Temporal Gyrus; THA: Thalamus;HC: Healthy Controls; CHR-P: Clinical High-risk Psychosis; FEP: First-episode Psychosis.

Supplementary Table 2.M100 amplitude in the passive condition.


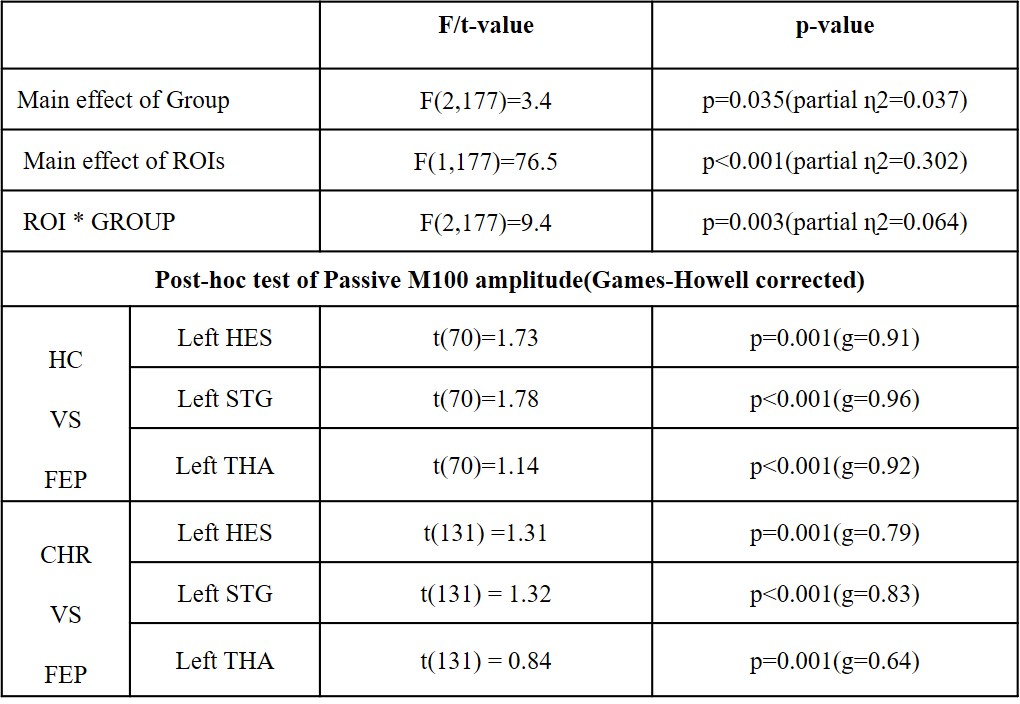


Abbreviations: HES: Heschl’s Gyrus; STG: Superior Temporal Gyrus; THA: Thalamus; HC: Healthy Controls; CHR-P: Clinical High-risk Psychosis; FEP: First-episode Psychosis.

In addition, we also explored the effects of antipsychotic medication (APMs) on the SAP effect in the FEP group. There were no significant differences between the medicated FEP group (n= 12) and medication-naïve FEPs in the 7 ROIs.
